# Promotion of Colitis in B Cell-Deficient C57BL/6 Mice Infected with Enterotoxigenic *Bacteroides fragilis*

**DOI:** 10.3390/ijms25010364

**Published:** 2023-12-27

**Authors:** Minjeong Jo, Soonjae Hwang, Chang-Gun Lee, Ju-Eun Hong, Da-Hye Kang, Sang-Hyeon Yoo, Woo-Seung Kim, Jung-Yoon Yoo, Ki-Jong Rhee

**Affiliations:** 1 Department of Biomedical Laboratory Science, College of Software and Digital Healthcare Convergence, Yonsei University Mirae Campus, Wonju 26493, Republic of Korea; jominjeong@skku.edu (M.J.); soonjae@gachon.ac.kr (S.H.); cglee@yonsei.ac.kr (C.-G.L.); jehong@yonsei.ac.kr (J.-E.H.); loolzeo@gmail.com (D.-H.K.); yshyyb@yonsei.ac.kr (S.-H.Y.); redberry1245@yonsei.ac.kr (W.-S.K.); jy_yoo@yonsei.ac.kr (J.-Y.Y.); 2 Department of Molecular Cell Biology, Sungkyunkwan University School of Medicine, Suwon 16419, Republic of Korea; 3 Department of Biochemistry, Lee Gil Ya Cancer and Diabetes Institute, College of Medicine, Gachon University, Incheon 21999, Republic of Korea

**Keywords:** enterotoxigenic *Bacteroides fragilis*, intestine, inflammation, IL-17A, B lymphocyte, colitis

## Abstract

Enterotoxigenic *Bacteroides fragilis* (ETBF) causes colitis and is implicated in inflammatory bowel diseases and colorectal cancer. The ETBF-secreted *B. fragilis* toxin (BFT) causes cleavage of the adherence junction, the E-cadherin, resulting in the large intestine showing IL-17A inflammation in wild-type (WT) mice. However, intestinal pathology by ETBF infection is not fully understood in B-cell-deficient mice. In this study, ETBF-mediated inflammation was characterized in B-cell-deficient mice (muMT). WT or muMT C57BL/6J mice were orally inoculated with ETBF and examined for intestinal inflammation. The indirect indicators for colitis (loss of body weight and cecum weight, as well as mortality) were increased in muMT mice compared to WT mice. Histopathology and inflammatory genes (*Nos2*, *Il-1β*, *Tnf-α*, and *Cxcl1*) were elevated and persisted in the large intestine of muMT mice compared with WT mice during chronic ETBF infection. However, intestinal IL-17A expression was comparable between WT and muMT mice during infection. Consistently, flow cytometry analysis applied to the mesenteric lymph nodes showed a similar Th17 immune response in both WT and muMT mice. Despite elevated ETBF colonization, the ETBF-infected muMT mice showed no histopathology or inflammation in the small intestine. In conclusion, B cells play a protective role in ETBF-induced colitis, and IL-17A inflammation is not attributed to prompted colitis in B-cell-deficient mice. Our data support the fact that B cells are required to ameliorate ETBF infection-induced colitis in the host.

## 1. Introduction

The gut microbiota plays a significant role in intestinal inflammation and carcinogenesis. The gram-negative bacterium *Bacteroides fragilis* is a human colonic symbiont [1,2]. Among the *B. fragilis* strains, *B. fragilis* toxin (BFT)-secreting *B. fragilis* is called enterotoxigenic *Bacteroides fragilis* (ETBF). Enterotoxigenic *Bacteroides fragilis* (ETBF) is strongly related to the development of inflammatory bowel disease (IBD) and colorectal cancer (CRC). ETBF is widely detected in biopsy specimens from the colonic contents of patients with IBD [3,4] and CRC [5,6].

Canonically, the metalloprotease toxin termed BFT, which is produced by ETBF, has been reported to be required for ETBF-induced colitis [7] and colonic tumorigenesis [8]. ETBF infection-induced inflammation could also promote colonic tumorigenesis in *APC*^Min+/−^ mice, which is dependent on the interleukin (IL) 17 response [8], as indicated by data showing down-regulation of ETBF-mediated tumorigenesis when IL-17A is neutralized by antibody or genetic abrogation in vivo [9,10].

Our group also demonstrated that ETBF infection accelerated azoxymethane (AOM)/DSS-induced carcinogenesis in C57BL/6 mice via active BFT [11]. BFT induces E-cadherin cleavage, which disrupts the colonic epithelial barrier and triggers an inflammatory response [12,13,14]. BFT-induced ectodomain cleavage of E-cadherin triggers the nuclear translocation of β-catenin, which activates nuclear factor-κB (NF-κB) to promote cellular proliferation and the production of inflammatory cytokines such as CXCL1 [15,16]. Nevertheless, the underlying mechanisms of ETBF infection-induced colitis remain unclear.

Although a T cell-dependent tumorigenic mechanism was investigated by other investigators [8,9,17], the role of B cells in the immune response against ETBF-induced colitis has been poorly studied. Other studies showed that B cells are essential for protective immunity to infectious pathogens [18,19]. The *Citrobacter rodentium* (*C. rodentium*) mouse enteritis model is characterized by IL-17A inflammation [20] and is commonly used to study enteropathogenic *Escherichia coli* (EPEC), as EPEC does not fully colonize the mouse intestine [21,22]. *C. rodentium* is the only known murine A/E pathogen [21,22]. Clearance of *C. rodentium* requires 4–6 weeks in WT mice. However, that clearance process is delayed in B-cell-deficient muMT mice, which also show increased colitis by *C. rodentium*. However, ETBF infection-induced colitis and the immune response in B-cell-deficient mice have not been explored. Previously, murine BFT-specific antibodies were not detected in C57BL/6 mice infected with ETBF [13], which led to the hypothesis that murine B cells are incapable of producing BFT-specific antibodies due to the structural similarity of BFT to host matrix metallopeptidase proteins [23]. However, there have been no studies on the effect of a deficiency of antibodies against BFT or on the antibodies’ role in the context of the development of ETBF-mediated colitis and the process of host defense.

In light of these data, we hypothesized that ETBF-induced colitis will be exacerbated in B-cell-deficient mice, as B cell depletion enhances susceptibility to infectious microbes [24,25]. In the current study, we investigate ETBF-induced colitis using B-cell-deficient muMT C57BL/6 mice. We assessed ETBF-induced colitis in muMT C57BL/6J mice and provide evidence that B cells are required for the host immunity to ETBF-mediated colitis. Our data suggest the hypothesis that asymptomatic ETBF colonization in humans might be dependent on the immunological characteristics of the host.

## 2. Results

### 2.1. ETBF Infection Increases Indirect Parameters of Colon Inflammation in B-Cell-Deficient Mice

**Figure 1:**
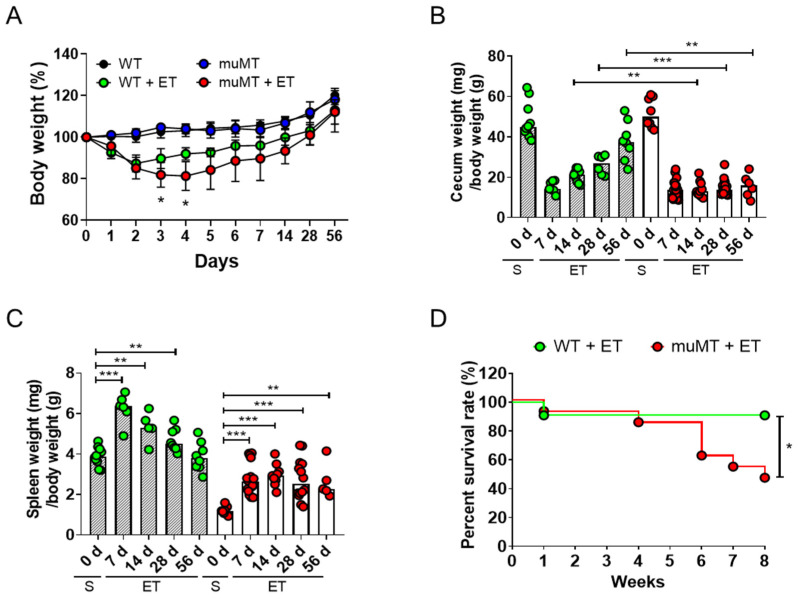
Clinicopathological parameters of ETBF-infected mice. Wild-type (WT) and muMT mice were infected with ETBF (1 × 10^9^ CFU), and indirect inflammation parameters were assessed. (**A**) Effect of ETBF infection on body weight. The body weights of individual mice were normalized to the starting body weight. * *p* < 0.05 vs. ETBF-matched, genotype control. Mann–Whitney test. (**B**) Effect of ETBF infection on cecal weight (mg)/ body weight (g) ratio. ** *p* < 0.01, *** *p* < 0.001, Mann–Whitney test. Each dot represents one mouse. The bar graph represents the median. (**C**) Effect of ETBF infection on the spleen weight (mg)/ body weight (g) ratio. ** *p* < 0.01, *** *p* < 0.001, Mann–Whitney test. (**D**) Kaplan–Meier curves depicting the survival rates after ETBF infection. Results are pooled from three independent experiments (*n* = 7–15 mice per group). S, sham. ET, ETBF. * *p* < 0.05, Mantel–Cox log-rank test.

ETBF-infected mice exhibit body weight loss as a result of colitis [13]. Moreover, decreased cecum weight and splenomegaly are positively associated with the degree of colitis in ETBF-infected mice [13]. To determine whether B lymphocytes protect colonic inflammation from ETBF, WT and muMT (B-cell-deficient mice) mice were infected with ETBF, separately, and indirect indicators of colitis were assessed. The ETBF-infected WT mice exhibited decreased body weight on day 2 and recovered their initial body weights by day 7 (Figure 1A). The ETBF-infected muMT mice showed a similar decrease in body weight on day 2 but had not fully recovered their initial body weight by day 28. The cecum/body weight ratio of the ETBF-infected WT mice was lowest on day 7 and then gradually recovered (Figure 1B). In contrast, this ratio was not restored in ETBF-infected muMT mice, and it remained low until at least day 56. The ceca of ETBF-infected muMT mice were devoid of cecal contents, and in some mice, they contained blood clots. In the ETBF-infected WT mice, the spleen/body weight ratio was increased on day 7, and it steadily returned to its original ratio by day 56 (Figure 1C). However, the ETBF-infected muMT mice maintained an increased spleen/body weight ratio for the duration of the experiment. Moreover, chronic bacterial colonization did not significantly affect the survival of ETBF-infected WT mice, but it dramatically reduced the survival of ETBF-infected muMT mice (Figure 1D). These results all indicate that muMT mice are dramatically more susceptible to ETBF infection than WT mice.

### 2.2. Persistent Inflammation in Large Intestines of ETBF-Infected B-Cell-Deficient Mice

**Figure 2:**
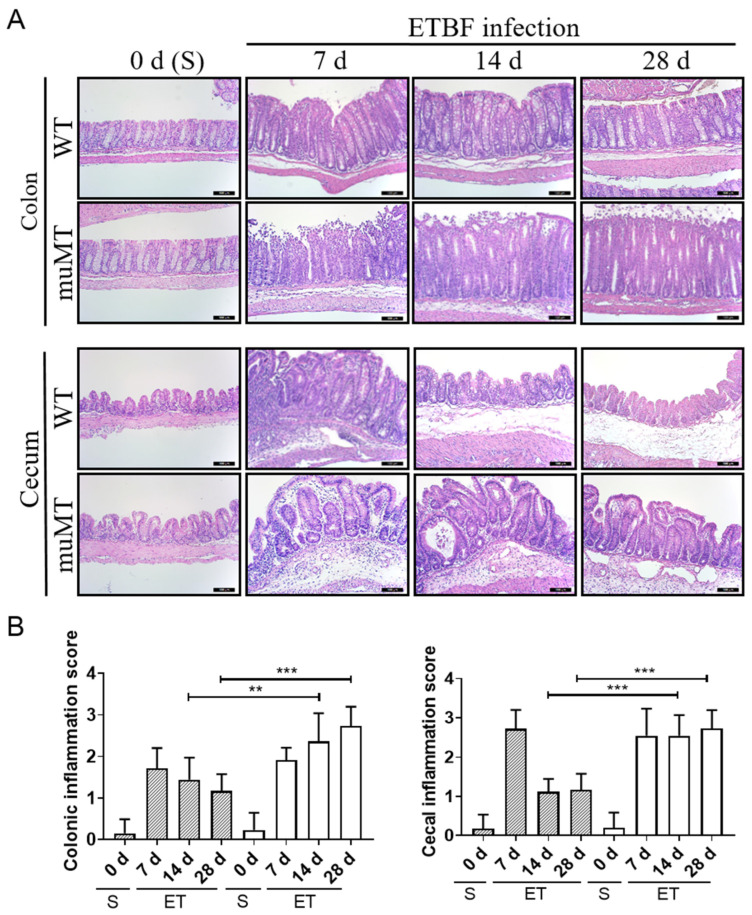
H&E staining of the large intestines of ETBF-infected mice. Wild-type (WT) and muMT mice were infected with ETBF, and FFPE tissues (colon and cecum) were stained with H&E. (**A**) H&E-stained colon and cecum tissues. Representative images are shown. Magnification ×200. Bar, 100 mm. (**B**) Inflammation scores for colons and ceca. The scores were assessed based on the criteria shown in Supplementary Table S1. Five representative fields from each mouse were assessed to determine the inflammation score. Five mice from each group were examined. Data are expressed as the mean ± SEM. S, sham. ET, ETBF. ** *p* < 0.01, *** *p* < 0.001, Mann–Whitney test.

To directly assess histological damage, the large intestines (cecum and colon) of ETBF-infected mice were analyzed for histological inflammation via hematoxylin and eosin (H&E) staining. The ETBF-infected WT mice showed marked crypt hyperplasia, increased mitotic activity in the colonic epithelium, and inflammatory cell infiltration with rounding and detachment of enterocytes on day 7 (Figure 2A, upper panels; Figure 2B). The colonic inflammation gradually decreased until day 28 (Figure 2A). In contrast, ETBF-infected muMT mice exhibited prominent crypt hyperplasia, increased mitotic activity in the colonic epithelium, and inflammatory cell infiltration with rounding and detachment of enterocytes from day 7 to day 28 in the colon. Similarly, the ceca of ETBF-infected WT mice showed ulcerations, extensive inflammatory cell infiltration, and in some mice, abscesses on day 7, but they then returned to near-normal levels by day 14–28 (Figure 2A, lower panels; Figure 2B. In contrast, the ETBF-infected muMT mice showed increased ulceration, inflammatory cell infiltration, and abscesses until day 14–28. In addition, the muMT mice showed 10-fold higher ETBF colonization than the ETBF-infected WT mice (Supplementary Figure S1). Taken together, the histological data suggest that the ETBF-infected muMT mice showed aggravated colitis compared with the WT mice.

### 2.3. Elevated Inflammatory Mediators in Cecum of B-Cell-Deficient Mice

**Figure 3:**
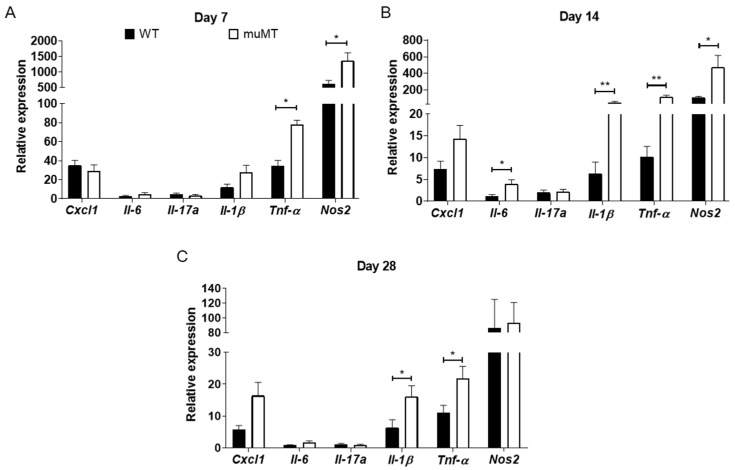
Expression of pro-inflammatory mediators in ceca of ETBF-infected mice. Wild-type (WT) and muMT mice were infected with ETBF (1 × 10^9^ CFU). Ceca were analyzed by qRT-PCR for expression of pro-inflammatory mediators on (**A**) day 7, (**B**) day 14, and (**C**) day 28 post-infection. GAPDH was used to normalize expression between different RNA samples. Relative expression was calculated using the comparative 2^−∆∆Ct^ method. Data are expressed as the mean ± SEM. * *p* < 0.05, ** *p* < 0.01, Mann–Whitney test. *n* = 5–10 mice per group.

As ETBF-infected muMT mice showed increased histologic inflammation of the large intestine compared with ETBF-infected WT mice, we determined which inflammatory mediators were elevated using qRT-PCR. Compared with ETBF-infected WT mice, ETBF-infected muMT mice showed higher expression levels of TNF-α and NOS2, but there were no differences in CXCL1, IL-6, IL-17A, or IL-1β expression on day 7 (Figure 3A). The higher expression levels of TNF-α and NOS2 in the ETBF-infected muMT mice compared with the ETBF-infected WT mice were sustained on day 14 (Figure 3B). In addition, ETBF-infected muMT mice exhibited increased IL-6 and IL-1β expression compared with ETBF-infected WT mice on day 14, whereas CXCL1 and IL-17A showed no statistical differences between the two groups. The IL-1β and TNF-α expression was higher in the ETBF-infected muMT mice than in the ETBF-infected WT mice until day 28 (Figure 3C), at which time CXCL1, IL-6, IL-17A, and NOS2 expression was comparable between the two groups. Overally, qRT-PCR analysis of the cecum showed that the expression of most pro-inflammatory genes was increased in ETBF-infected muMT mice compared to WT mice.

### 2.4. ETBF Does Not Induce Inflammation in the Small Intestine

**Figure 4:**
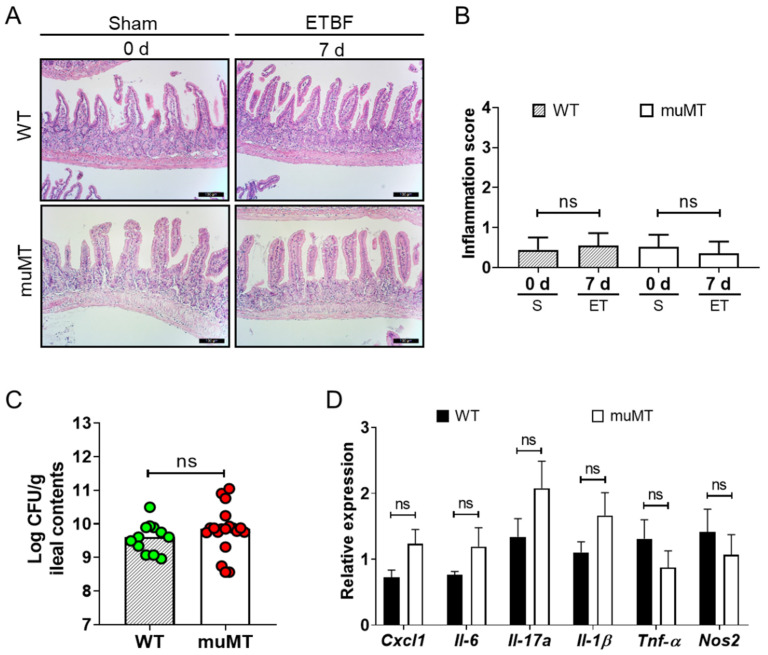
H&E staining and expression of pro-inflammatory mediators in the ilea of ETBF-infected mice. Wild-type (WT) and muMT mice were infected with ETBF (1 × 10^9^ CFU) for 7 days. (**A**) Ilea from ETBF-infected and uninfected WT and muMT mice were stained with H&E on day 7. Representative images are shown. Magnification ×200. Bar, 100 mm. (**B**) Inflammation scores in ilea on day 7. Five representative fields from each mouse were assessed to determine the inflammation scores. Five mice from each group were examined. Data are expressed as the mean ± SEM. S, sham. ET, ETBF. (**C**) ETBF colonization was assessed by bacterial culture of the ileal contents on day 7. Each dot represents one mouse. The bar graph represents the median. (**D**) Ilea were analyzed for expression of pro-inflammatory mediators by qRT-PCR on day 7. GAPDH was used to normalize expression between different RNA samples. The relative expression was calculated by the comparative 2^−∆∆Ct^ method. Data are expressed as the mean ± SEM. Mann–Whitney test. ns, not significant.

The increased mortality and higher ETBF colonization observed in the ETBF-infected muMT mice led us to investigate whether the small intestine was also damaged by ETBF infection. The ilea of the small intestines were histologically examined via H&E staining. However, ilea showed no histologic evidence of inflammation in either the ETBF-infected WT mice or the ETBF-infected muMT mice (Figure 4A,B), although ETBF colonization was detected in the ileal contents at levels comparable to the colonic contents on day 7 (Figure 4C, Supplementary Figure S1). Consistent with the histology, we found no differences in the expression of pro-inflammatory mediators (CXCL1, IL-6, IL-17A, IL-1β, TNF-α, and NOS2) between the ETBF-infected WT and muMT mice and the uninfected control mice (Figure 4D). These results suggest that ETBF colonization does not induce inflammation in the small intestines of muMT mice.

### 2.5. Th17/γδ T and Treg Response in ETBF-Infected B-Cell-Deficient Mice

**Figure 5:**
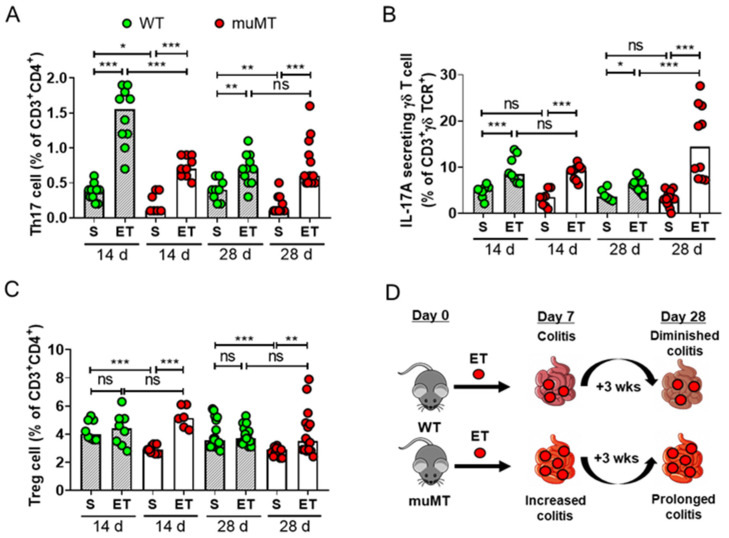
Th17, IL-17A-secreting γδ T cells, and Treg cells in ETBF-infected mice. Wild-type (WT) and muMT mice were infected with ETBF (1 × 10^9^ CFU) and examined on day 14 or day 28. The proportions of Th17, γδ T cells, and Treg cells in the mesenteric lymph nodes were measured by flow cytometry. (**A**) Percentage of Th17 cells (CD3^+^, CD4^+^, IL-17A^+^). (**B**) Percentage of IL-17A-secreting γδ T cells (CD3^+^, γδ TCR^+^, IL-17A^+^). (**C**) Percentage of Treg cells (CD3^+^, CD4^+^, CD25^+^, FoxP3^+^). S, sham. ET, ETBF. * *p* < 0.05, ** *p* < 0.01, *** *p* < 0.001, Mann–Whitney test. Each dot represents one mouse. The bar graph represents the median. (**D**). A diagram depicting the changes in ETBF-infected WT and muM mice. In WT mice, colonic inflammation reached peak at 7 days post-infection and then gradually decreased by 28 days. In muMT mice, extensive colonic inflammation is maintained up to 28 days. Colonic ETBF numbers are increased in muMT mice.

ETBF colonization induces a Th17 response in mice that is characterized by an increased number of IL-17A-producing Th17 cells and γδ T cells [8]. In addition, ETBF-mediated colitis persists, leading to synchronized expansion of IL-17A-producing inflammatory Th17 cells/γδ T cells and immune-suppressive FoxP3^+^ T-cells (Tregs) in the large intestine of mice [9]. The IL-17A-producing cell/Treg balance is suggested to determine the outcome of T-cell-dependent inflammation [26]. To investigate the role of B cells in the ETBF-induced IL-17A response, we assessed the ratio of Th17/γδ T cells and Tregs in the mesenteric lymph nodes (MLNs) of ETBF-infected mice via flow cytometry. The data showed that the ratio of Th17 cells in the MLNs increased on day 14 in both ETBF-infected WT and ETBF-infected muMT mice compared with the non-infected control mice (Figure 5A, Supplementary Figure S2). The high levels of Th17 cells were sustained until day 28 in the ETBF-infected muMT mice, whereas the levels had decreased by day 28 in the ETBF-infected WT mice. The ratio of IL-17A-secreting γδ T cells was elevated in both the ETBF-infected WT and ETBF-infected muMT mice compared with the non-infected control mice (Figure 5B). The number of IL-17A-secreting γδ T cells did not differ between the ETBF-infected WT mice and ETBF-infected muMT mice on day 14, but it was higher in the ETBF-infected muMT mice on day 28. The numbers of Treg cells did not increase in the ETBF-infected WT mice compared with the non-infected WT mice on day 14 or day 28 (Figure 5C). In contrast, the ETBF-infected muMT mice showed increased Treg cell levels on both day 14 and day 28 compared with non-infected muMT mice. However, the number of Treg cells did not differ between the ETBF-infected WT mice and the ETBF-infected muMT mice. These results indicate that the populations of IL-17A-secreting Th17/γδ T cells were increased during ETBF infection. The increased IL-17A-secreting Th17/γδ T cells were sustained up to day 28 in ETBF-infected muMT mice, whereas IL-17A-secreting Th17/γδ T cells decreased at day 28 in ETBF-infected WT mice. However, the expression level of the IL-17A gene was comparable between muMT mice and WT mice during ETBF infection (Figure 3B,C). Collectively, we suggest that exacerbated colitis in ETBF-infected muMT mice was not contributed by Th17 immune response.

## 3. Discussion

ETBF has emerged as an opportunistic pathogen that triggers an imbalance in intestinal health [5]. Several studies have shown that ETBF infection in mice induces a predominant intestinal Th17 response that promotes colitis [8,27,28]. However, the role of B lymphocytes has remained elusive in the pathogenesis of ETBF-mediated colitis. The results presented here are the first study to investigate ETBF-induced colitis using B-cell-deficient mice.

In muMT mice, ETBF infection-induced colitis was prolonged and more lethal than in ETBF-infected WT mice. One contributing factor could be that the ETBF-infected muMT mice had 10-fold higher colonization in the large intestine compared with ETBF-infected WT mice. A plausible explanation for the higher colonization is that the muMT mice lack antibodies that are known to control bacterial colonization levels. It has been reported that bacterial surface molecules, such as bacterial polysaccharides, induce IgG production [29]. When BALB/c mice were infected with *E. coli* O157 and injected intraperitoneally with IgG, the intestinal pathology decreased compared with *E. coli* O157-infected mice non-treated with IgG [30]. Furthermore, bacterial colonization was lower in the IgG-treated mice than in the non-treated mice. More importantly, the gut mucosa secretes IgA specialized for mucosal protection against infection to maintain bacteria–host homeostasis [31,32]. Intestinal IgA responses to microbes interfere with the adherence of gut bacteria to epithelial cells and neutralize bacterial toxins [33]. The secretory IgA produced in the intestinal tract can bind to both *E. coli* O157 membrane macromolecules and secreted proteins [34]. Based on those reports, it is likely that the lack of B cells, and thus antibodies, in the muMT mice is the cause of higher bacterial colonization and aggravated colitis.

Gut microbiota and metabolites are important in intestinal homeostasis. *B. fragilis* produces short-chain fatty acids (SCFAs), including propionate, acetate, and butyrate, from dietary fiber fermentation [35]. It was reported that a decrease in the concentration of SCFAs promotes IBD [36]. In addition, it was reported that SCFAs modulate the cecal lamina propria to decrease IL-17A-producing γδ T cells, although IL-17A-producing αβ T cells were unaffected [37]. Propionate regulates γδ T cells, resulting in the downregulation of IL-17A production in patients with IBD [38]. In an animal model of TNBS-induced colitis, butyrate suppressed IL-17A production by disrupting the Treg/Th17 balance [39]. We found that the IL-17A expression level in intestinal tissues was similar in ETBF-infected muMT mice and ETBF-infected WT mice. Nevertheless, as the number of IL-17A-secreting γδ T cells in ETBF-infected muMT mice was higher than in ETBF-infected WT mice, gut microbiota-derived SCFA might suppress the production of IL-17A cytokine from γδ T cells. As *IL-1β*, *Tnf-α,* and *Nos2* expression was elevated in ETBF-infected muMT mice compared with ETBF-infected WT mice, the up-regulation of innate immune responses in ETBF-infected muMT mice could contribute to the persistent colitis in B-cell-deficient mice, which needs further study.

The production of naive CD4^+^ T cells is reported to be similar in both WT mice and muMT mice [40,41]. In the current study, we found that uninfected muMT mice exhibited lower percentages of both Th17 cells and Treg cells than uninfected WT mice. The differences in the baseline levels of Th17 cells and Treg cells could be multifactorial, but several reports have suggested that B cells can promote the production of Treg cells. The reduced percentage of Treg cells in non-infected muMT mice has been shown previously [42,43,44]. Ray et al. found that muMT mice as well as anti-CD20-depleted B-cell-deficient mice showed a reduction in peripheral Treg cells [45]. Furthermore, adoptive transfer of B cells into muMT mice restored Treg cell numbers. The authors further showed data suggesting that glucocorticoid-induced TNF receptor family-related protein ligand (GITRL) expression by B cells and cognate interaction of GITR expressed on Treg cells could induce Treg cell proliferation, resulting in homeostatic numbers of Treg cells. In our study, the percentage of Treg cells was increased in ETBF-infected muMT mice compared with non-infected muMT mice. In stark contrast, the percentages of Treg cells remained constant in both ETBF-infected WT mice and non-infected WT mice. Without a more extensive analysis of various lymphocyte subsets, it is difficult to draw a conclusion at this time. A more simplistic explanation could lie in the tissues used in the current study. We used mesenteric lymph nodes, whereas a physiologically relevant tissue would be lamina proporia lymphocyte. These experiments are planned for future studies.

DBA/1J mice depleted of B cells using anti-CD20 antibodies showed a delay in collagen-induced arthritis and a decrease in T cell activation and proliferation [46,47,48]. Similarly, the lack of B cells in muMT mice could explain the differences from WT mice in both Th17 cells and Treg cells, which could in turn translate into different degrees of colitis between the two mouse types. Most IL-17A produced in the early phase of ETBF infection response was reported to depend on Th17 cells, and the late stage of the ETBF-induced inflammatory response was reported to depend on γδ T cells [8,13,49].

Another unexpected finding of this study is the lack of inflammation in the ileum of both ETBF-infected WT mice and ETBF-infected muMT mice, even though the ileum lumen harbored high levels of ETBF in both the WT and muMT mice. A possible mechanistic explanation is that ETBF does not secrete BFT in the ileum during infection, or that the ileum contains an antimicrobial factor that neutralizes the secreted BFT. Paneth cells exist only in the small intestine and secrete α-defensins that kill or inactivate microorganisms in response to bacterial products and pro-inflammatory cytokines [50,51]. α-defensins can neutralize several toxins in humans, including diphtheria toxin, *Pseudomonas aeruginosa* exotoxin A, and anthrax lethal toxin [52,53]. The reason for the lack of inflammation in the ilea of ETBF-infected mice is unclear. Further studies on the small intestine could shed light on the mechanism of colitis in ETBF-infected mice.

In summary, we found that B-cell-deficient mice infected with ETBF showed exacerbated colitis compared with ETBF-infected WT mice. Colonization with ETBF led to the promoted induction of Th17 cells in infected WT mice vs. that in the uninfected control mice; meanwhile, Th17 immune response was delayed in ETBF-infected muMT mice. However, IL-17A expression was not increased in ETBF-infected muMT mice compared with ETBF-infected WT mice. We suggest that although mucosal B cells are initially required to ameliorate ETBF-mediated colitis, they do not affect the IL-17A response to ETBF colitis.

## 4. Materials and Methods

### 4.1. Bacterial Strains

The wild-type enterotoxigenic *Bacteroides fragilis* (ETBF) strain, *B. fragilis* 86-5443-2-2 (*bft*-2), is naturally resistant to clindamycin and gentamicin. ETBF was grown in brain heart infusion (BHI) broth and BHI agar supplemented with L-cysteine (Sigma-Aldrich, St. Louis, MO, USA), hemin (Sigma-Aldrich, St. Louis, MO, USA), clindamycin (Hospira, Chicago, IL, USA), and gentamicin (Corning Incorporated, Corning, NY, USA) for 2 days at 37 °C under anaerobic conditions using a Pack-Anaero (Mitsubishi Gas Chemical CO. Inc., New York, NY, USA).

### 4.2. Mouse Experiments

Specific-pathogen-free, 6-week-old female C57Bl/6J mice were purchased from Jackson Laboratories (Bar Harbor, ME, USA). Female or male B6.129S2-*Ighm^tm1Cgn^*/J (muMT) mice deficient in mature B lymphocytes (backcrossed to C57Bl/6 background at least 10 times) were originally purchased from Jackson Laboratories (Bar Harbor, ME, USA), maintained by homozygous mating, and bred in the animal facility at Yonsei University (Wonju, South Korea). Both the WT mice and muMT mice used in these studies were housed in individually HEPA-filtered cages with a 12 h light–dark cycle and given ad libitum access to standard diet and water. All mice received antibiotic water containing clindamycin (100 mg/L) and gentamicin (300 mg/L) 5 days before bacterial inoculations, which continued for the duration of the experiments to promote *B. fragilis* colonization. Bacteria were suspended in PBS (Welgene, Gyeongsan-si, Gyeongsangbuk-do, South Korea), and 1 × 10^9^ CFU/200 μL were administered through oral gavage. Colonization of bacteria was monitored by 10-fold serial dilution of stool and plating in BHI agar containing clindamycin (6 μg/mL) and gentamicin (50 μg/mL). Characteristic *B. fragilis* colonies were enumerated after anaerobic culture and are reported as CFU/gram stool. All mice harbored the inoculated ETBF at approximately 1 × 10^9^ CFU/gram stool during the experiments. All animal housing and experimental procedures were approved by the Institutional Animal Care and Use Committee (IACUC) and Institutional Biosafety Committee (IBC) of Yonsei University MIRAE Campus, in accordance with the regulations of the Association for the Assessment and Accreditation of Laboratory Animal Care International (#YWCI-201608-006-01, #201512-P-004-01).

### 4.3. Hematoxylin and Eosin (H&E) Staining

Mice were euthanized by CO_2_ asphyxiation, and their colons and ceca were excised. The colons were incised in the longitudinal direction, washed with PBS, and placed flat on a plate for fixation with 10% formalin (Merck, Kenilworth, NJ, USA). The colon was shaped into a Swiss roll. The ceca were fixed with 10% formalin after removing the cecal contents. The colons and ceca were embedded in paraffin (Merck, Kenilworth, NJ, USA) and sectioned at 4 μm with a rotary microtome (Leica, Wetzlar, Germany). The tissues on slides were then stained with hematoxylin, bluing solution, and eosin for 8 min, 4 min, and 30 s, respectively. The stains were photographed by optical microscopy (Leica, Wetzlar, Germany) and rendered using Leica software (version 5.1.0).

### 4.4. Histological Evaluation of Inflammation in the Colon and Ileum

The large intestines were excised and divided into the cecum, proximal colon, and distal colon. The cecum was longitudinally divided in half. The first half of the cecum was fixed in 10% formalin, and the second half of the cecum frozen in liquid nitrogen. The proximal and distal colons were Swiss-rolled and fixed in 10% formalin. The sections were stained with H&E and histologically assessed for epithelial damage. The extent of colon tissue inflammation was evaluated based on the extent of ulceration, crypt abscess, erosion, hyperplasia, and immune cell infiltration in the colon. The extent of ileum tissue inflammation was evaluated based on epithelial hyperplasia, the extent of inflammatory cell infiltration in the mucosa and lamina propria, and crypt loss. The histopathological colitis and ileitis scores derived from these criteria are shown in Supplementary Table S1. Each histologic parameter was assessed based on the features in Supplementary Table S1 to indicate the specific degree of each lesion, and the average of all parameters was used to determine the total inflammation score. Five representative fields from each mouse were assessed to assign an inflammation score. Five mice from each group were examined.

### 4.5. Bacterial Colonization

Briefly, a 10-fold serially diluted stool sample was inoculated on BHI agar. The plates were grown for 2 days at 37 °C under anaerobic conditions (Pack-Anaero; Mitsubishi Gas Chemical Co., Inc., New York, NY, USA) in BHI agar supplemented with hemin and cysteine. BHI agar was supplemented with clindamycin and gentamicin to inhibit growth of other bacteria and promote colonization of the *Bacteroides* strains. *B. fragilis* colonies are phenotypically recognizable as opaque and domed, compared with other opportunistic colonies. *B. fragilis* was absent in the feces of uninfected mice.

### 4.6. RNA Extraction

Total RNA was extracted from tissues using TRIzol (Ambion, Austin, TX, USA). Pieces of distal colon tissues were treated with 800 μL of TRIzol reagent, homogenized using a pestle, and incubated at room temperature (RT) for 10 min. Then, 200 μL of chloroform was added and centrifuged at 12,000× *g*, 4 °C for 15 min. The upper layer was transferred to a microfuge tube, and an equal amount of isopropanol was added. After 10 min of incubation at RT, the mixture was centrifuged at 12,000× *g*, 4 °C for 10 min. Then, the supernatant was removed, and 1 mL of 75% ethanol was added. Finally, the mixture was centrifuged at 7500× *g*, 4 °C for 5 min, and the supernatant was removed. The RNA pellet was air-dried and dissolved in 20 μL of diethylpyrocarbonate-treated water (Invitrogen, Carlsbad, CA, USA). RNA quantity and quality were examined using an Infinite M200 PRO TECAN (Research Triangle Park, NC, USA). The isolated total RNA was stored at −80 °C until use.

### 4.7. Quantitative Real-Time PCR (qRT-PCR)

The concentration of RNA was adjusted to 5 μg/μL for cDNA synthesis using a random primer (Invitrogen, Carlsbad, CA, USA) and MMLV-reverse transcriptase (Invitrogen, Carlsbad, CA, USA). For RT-PCR analysis, mRNA was transcribed into cDNA using 40 cycles of denaturation for 3 s at 95 °C and annealing for 30 s at 60 °C in 20 µL reaction volumes using an ABI 7500 FAST Real-Time PCR system (Applied Biosystems, Foster City, CA, USA). qRT-PCR was performed with a TaqMan Gene Expression Assay. All probes were purchased from Thermo Fisher Scientific (Waltham, MA, USA). Gene expression was normalized using glyceraldehyde 3-phosphate dehydrogenase (GAPDH). Quantitation and analysis of relative gene expression were performed by the 2^−∆∆Ct^ method as described by Livak and Schmittgen [54]. The sham groups were arbitrarily assigned a value of 1, and the infected groups were calculated relative to the relevant sham group. qRT-PCR was performed in triplicate. Probes for the qRT-PCR based on the TaqMan assay are summarized in Supplementary Table S2.

### 4.8. Flow Cytometry

Mouse mesenteric lymph nodes (MLNs) were collected, minced, and passed through a 40-μM nylon filter (Falcon, Franklin Lakes, NJ, USA) to acquire single cell suspensions. Cells were washed three times with 1 mL of Roswell Park Memorial Institute (RPMI) 1640 medium (Gibco, Grand Island, NY, USA) and cultured under stimulation conditions. Briefly, cells (1 × 10^7^) were treated with GolgiPlug (BD Biosciences, San Jose, CA, USA) containing phorbol myristate acetate (PMA), ionomycin, and brefeldin A for 4 h at 37 °C with 5% CO_2_. After incubation, the cells were stained with APC-Cy7-conjugated anti-CD3 ε monoclonal antibodies (clone: 145-2C11), PE-Cy7-conjugated anti-CD4 monoclonal antibodies (clone: RM4-5), and PE-CF594-conjugated anti-γδTCR (clone: GL3).

After surface staining, the cells were fixed and permeabilized. Then, the cells were stained with PE-conjugated anti-IL-17A monoclonal antibodies (clone: TC11-18H10) at 4 °C in the dark for 30 min to identify Th17 cells. After being washed three times with 1 mL of RPMI 1640 medium, the cells were stained with APC-Cy7-conjugated anti-CD3ε monoclonal antibodies (clone: 145-2C11), PE-Cy7-conjugated anti-CD4 monoclonal antibodies (clone: RM4-5), and BB515-conjugated anti-CD25 monoclonal antibodies (clone: PC61) at RT in the dark for 20 min. Then, the cells were fixed, permeabilized using Foxp3-specific buffers, and stained with Alexa 647-conjugated Foxp3 monoclonal antibodies (clone: MF23). All the antibodies and buffers were purchased from BD Biosciences (San Diego, CA, USA). Isotype control antibodies were used to confirm antibody specificity. Stained cells were analyzed by flow cytometry (BD Biosciences Pharmingen, San Diego, CA, USA). A representative dot plot analysis of the flow cytometric results is shown in Supplementary Figure S1.

### 4.9. Statistical Analysis

Median values were compared using the unpaired, two-tailed Mann–Whitney U test or chi-square test unless otherwise indicated. Kaplan–Meier survival curves were compared using the log-rank test. Statistical analyses were performed using GraphPad Prism 8 (GraphPad Software Inc., La Jolla, CA, USA). A *p*-value of <0.05 was considered to indicate a statistically significant difference.

## 5. Conclusions

In this study, we show that B cells play a protective role during ETBF-mediated colitis. Histological inflammation and expression of pro-inflammatory genes were increased in ETBF-infected B-cell-deficient mice. ETBF-induced intestinal inflammation is confined to the large intestine. However, ETBF infection-induced IL-17A inflammation was comparable between B-cell-deficient mice and WT mice. These results suggest that Th17 immune response is not a major etiology for exacerbated ETBF colitis in B-cell-deficient mice. Collectively, our results indicate that B cells suppress ETBF infection-induced colitis.

## Data Availability

The data presented in this study are available upon request from the corresponding author (K.-J.R.: kjrhee@yonsei.ac.kr).

## Supplementary Materials

The following supporting information can be downloaded at: https://www.mdpi.com/article/10.3390/ijms25010364/s1.
