# Supplementary material for: Promotion of Colitis in B Cell-Deficient C57BL/6 Mice Infected with Enterotoxigenic Bacteroides fragilis
Source: Int J Mol Sci. 2023 Dec 27;25(1):364. doi: 10.3390/ijms25010364 (PMC10778593; doi:10.3390/ijms25010364)
Supplement: Supplementary file 1 [file ijms-25-00364-s001.zip › ijms-2762786-supplementary.pdf]

[Supplementary data to *Int. J. Mol. Sci.*]

Promotion of Colitis in B-Cell-Deficient C57BL/6 Mice Infected with Enterotoxigenic *Bacteroides fragilis*

Minjeong Jo [1,2,†], Soonjae Hwang [1,3,†], Chang-Gun Lee [1], Ju-Eun Hong [1], Da-Hye Kang [1], Sang-Hyeon Yoo [1], Woo-Seung Kim [1], Jung-Yoon Yoo [1] and Ki-Jong Rhee [1,*]

1 Department of Biomedical Laboratory Science, College of Software and Digital Healthcare Convergence, Yonsei University Mirae Campus, Wonju 26493, Republic of Korea; jominjeong@skku.edu (M.J.); soonjae@gachon.ac.kr (S.H.); cglee@yonsei.ac.kr (C.-G.L.); jehong@yonsei.ac.kr (J.-E.H.); loolzeo@gmail.com (D.-H.K.); yshyyb@yonsei.ac.kr (S.-H.Y.); redberry1245@yonsei.ac.kr (W.-S.K.); jy_yoo@yonsei.ac.kr (J.-Y.Y.)

2 Department of Molecular Cell Biology, Sungkyunkwan University School of Medicine, Suwon 16419, Republic of Korea

3 Department of Biochemistry, Lee Gil Ya Cancer and Diabetes Institute, College of Medicine, Gachon University, Incheon 21999, Republic of Korea

* Correspondence: kjrhee@yonsei.ac.kr

† These authors contributed equally.

Supplementary Figure S1. ETBF colonization in colon in ETBF-infected mice.

Supplementary Figure S2. Flow cytometry dot plot of mesenteric lymph nodes in mice.

Supplementary Table S1. Histopathologic scoring of colon and ileum.

Supplementary Table S2. Probes for quantitative real-time PCR (TaqMan assay).

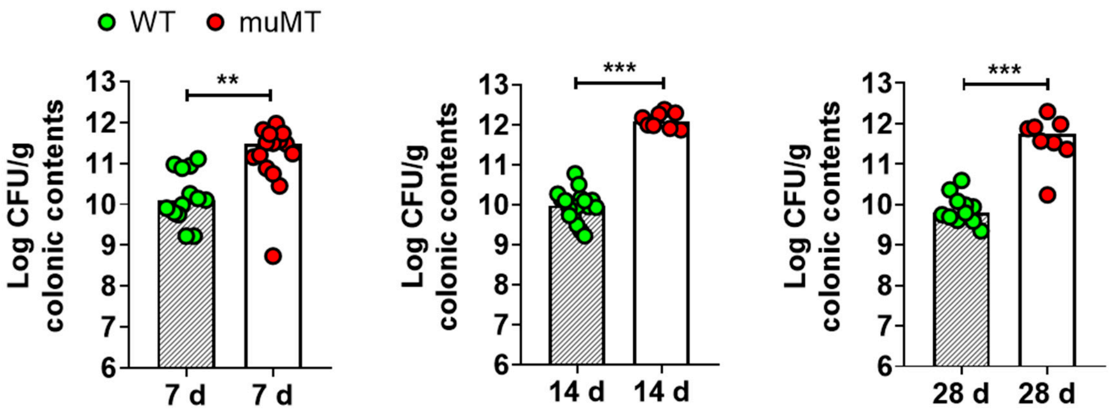

Supplementary Figure S1. ETBF colonization in colon of ETBF-infected mice.

Wild-type (WT) and muMT mice were infected with ETBF (1×10^9 CFU) for 28 days. ETBF colonization was assessed by bacterial culture of colonic contents at day 7, day 14 and day 28. **$p < 0.01$, ***$p < 0.001$, Mann-Whitney test. Each dot represents one mouse. The bar graph represents median.

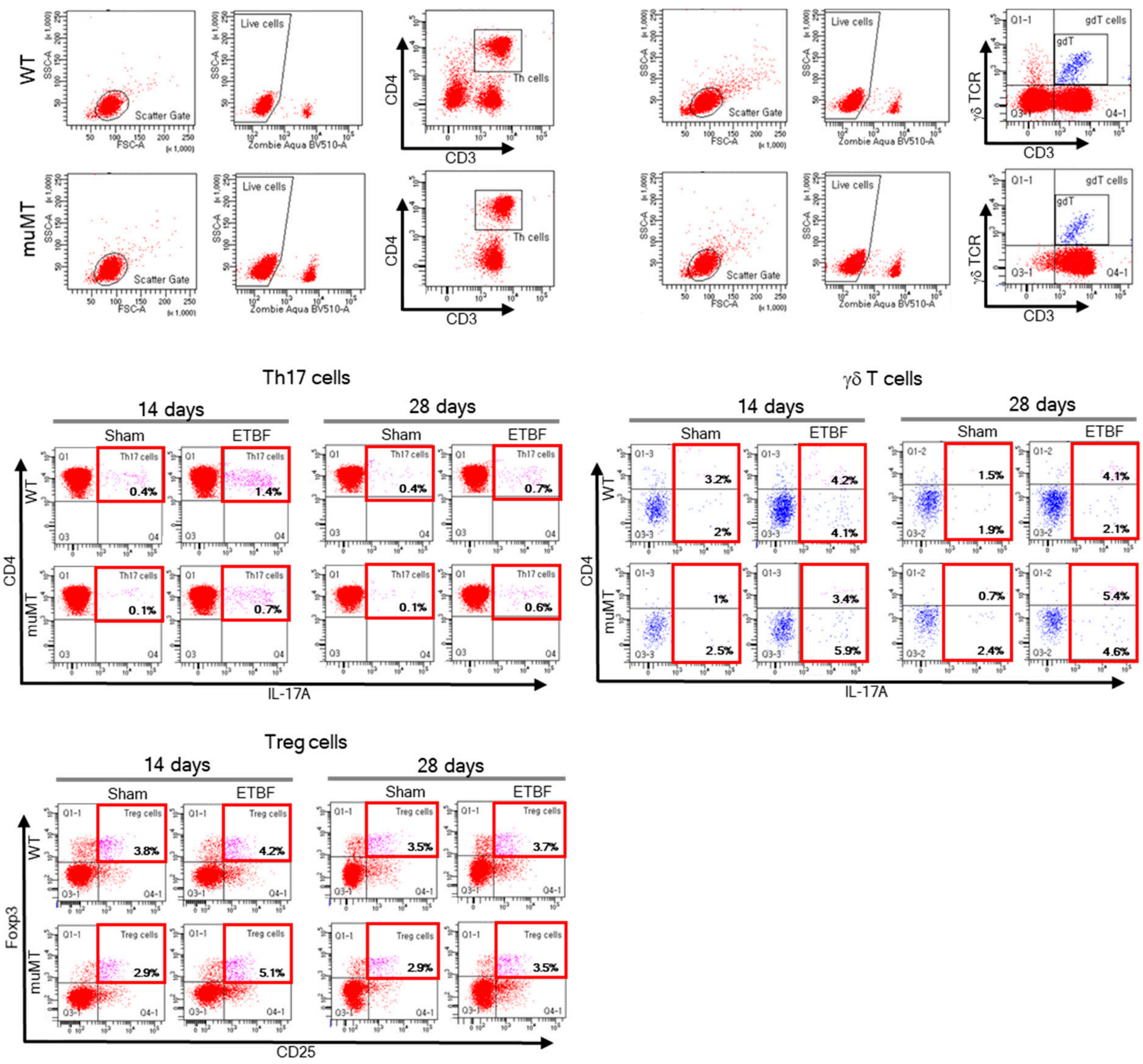

Supplementary Figure S2. Flow cytomeric dot plot of mesenteric lymph nodes in mice.

WT and muMT mice were infected with ETBF (1 × 10^9 CFU) for day 14 or day 28. The proportion of Th17, IL-17A secreting γδT and Treg cells in the mesenteric lymph nodes was measured by flow cytometry. Representative flow cytometry dot plots are shown.

Supplementary Table S1. Histopathology scoring of colon and ileum

Tissue	Category	Description	Score
Colon, Ileum	Normal	Intact epithelium<br>No crypt loss<br>85% < normal thickness of crypt length<br>No infiltration of neutrophils	0
	Mild	Involvement of the lamina propria<br>Crypt loss < 10%<br>70% < normal thickness of crypt length ≤ 85%<br>Mild infiltration of neutrophils	1
	Moderate	Involvement of the submucosa<br>10% ≤ crypt loss < 30%<br>50% ≤ normal thickness of crypt length < 70%<br>Moderate infiltration of neutrophils	2
	Severe	Transmural ulceration<br>30% < crypt loss<br>Normal thickness of crypt length < 50%<br>Severe infiltration of neutrophils	3

Supplementary Table S2. Probes for qRT-PCR (TaqMan assay)

Gene symbol	Assay ID
KC (Cxcl1)	Mm04207460_m1
Il-6	Mm00446190_m1
Il-17A	Mm00439618_m1
*Il-1-**β***	Mm00434228_m1
Tnf-a	Mm00443258_m1
Nos2	Mm00440502_m1
GAPDH	Mm99999915_g1
GPR35	Mm01973686_s1
